# Early life course processes leading to educational and economic attainment in young adulthood: Contributions of early socioeconomic adversity and education polygenic score

**DOI:** 10.1371/journal.pone.0256967

**Published:** 2021-10-11

**Authors:** Kandauda A. S. Wickrama, Catherine Walker OˋNeal, Tae Kyoung Lee, Seonhwa Lee

**Affiliations:** 1 Department of Human Development and Family Science, The University of Georgia, Athens, Georgia, United States of America; 2 Department of Public Health Sciences, University of Miami Miller School of Medicine, Miami, Florida, United States of America; 3 Department of Christian Studies, Seoul Women’s University, Seoul, Republic of Korea; University of Pennsylvania, UNITED STATES

## Abstract

The present study investigated an integrated life course model, drawn from the life course theoretical perspective, to elucidate youth’s additive, cascading, and cumulative life course processes stemming from early socioeconomic adversity and education polygenic score (education PGS) as well as potential interactions between them (GxE), which contribute to subsequent young adult socioeconomic outcomes. Additionally, the independent, varying associations among social and genetic predictors, life-stage specific educational outcomes (educational achievement in adolescence and educational attainment, in later stages), and young adult economic outcomes were examined. The study used prospective, longitudinal data from the National Longitudinal Study of Adolescent and Adult Health (Add Health) with a sample of 5,728 youth of European ancestry. Early family socioeconomic adversity and individual education PGS were associated with life stage-specific educational outcomes through *additive* and *cascading* processes linked to young adults’ economic outcomes (personal earnings) through a *cumulative* process. A GxE moderation existed between individuals’ education PGS and early socioeconomic adversity at multiple life stages, explaining variation in adolescent educational outcomes. Both early socioeconomic adversity and education PGS were persistently associated with youth’s educational and economic outcomes throughout the early life course. In sum, the findings based on the integrated life course model showed how additive, cascading, and cumulative processes were related and conditioned one another, generating specific life course patterns and outcomes. The findings highlight the value of incorporating molecular genetic information into longitudinal developmental life course research and provide insight into malleable characteristics and appropriate timing for interventions addressing youth developmental characteristics.

## Introduction

Developmental studies have documented the persistent association between childhood and adolescent socioeconomic adversity (hereafter referred to as early socioeconomic adversity) and youth educational and economic outcomes [1–3]. Genetic studies have also shown that measures capturing multiple genetic variants related to educational attainment (i.e., a polygenic score, PGS) explain variation in youth educational outcomes as well as economic outcomes [4]. Combining these two streams of research (i.e., research on socioeconomic adversity and genetic influences), we expect that both factors are a fundamental contributor to later well-being [5]. However, less is known about independent contributions of early socioeconomic adversity and education PGS on youth’s educational and economic outcomes over the early life course, encompassing adolescence, the transition to adulthood, and young adulthood [6].

**Fig 1:**
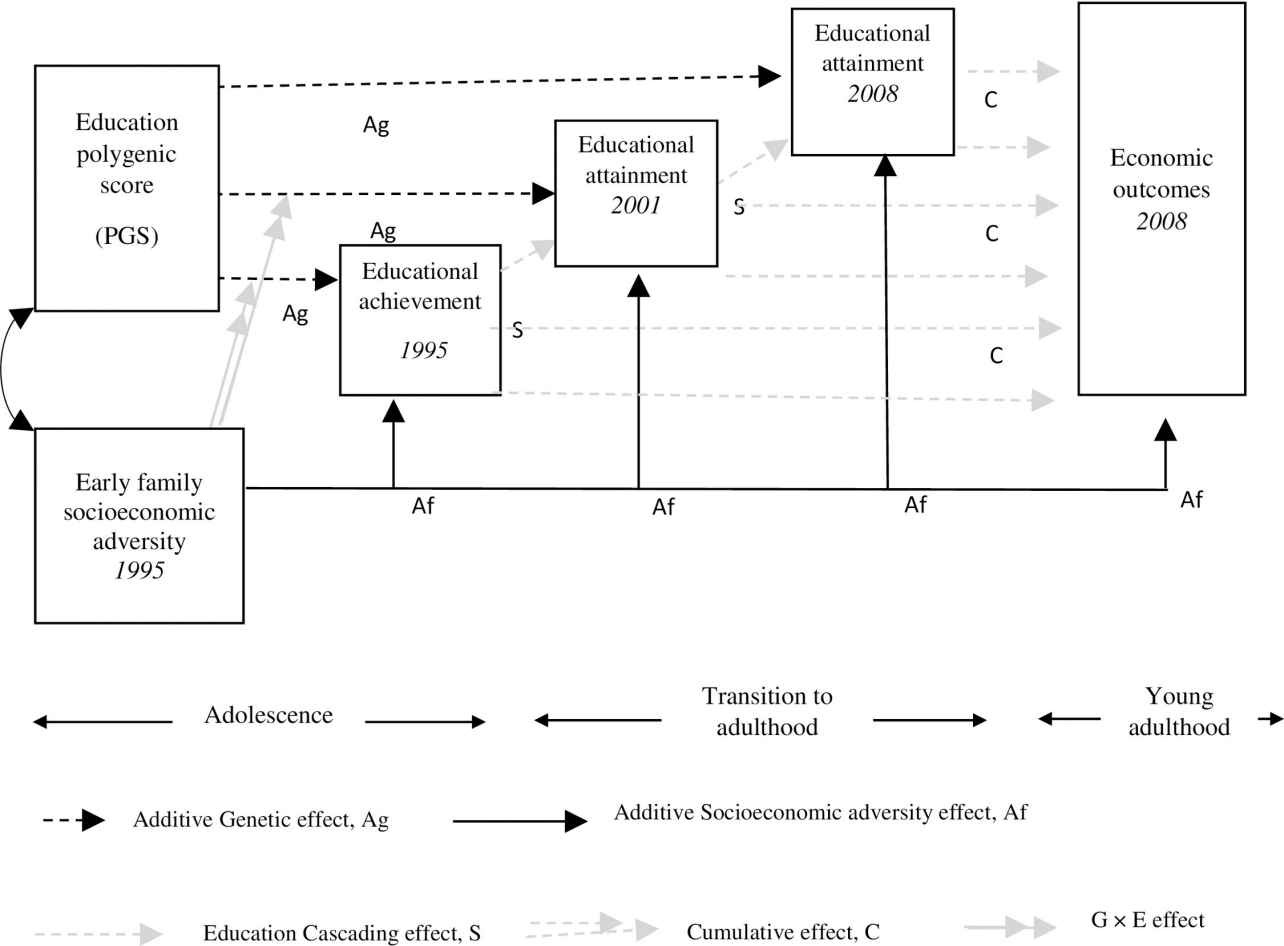
Early life course processes involving educational attainment leading to young adult economic outcomes.

As depicted in Fig 1, we posit that both early socioeconomic adversity and education PGS are persistently associated with youth’s educational and economic (i.e., personal earnings) outcomes in three distinct life course processes. First, as an *additive process*, each contributes independently to life-stage specific educational outcomes (i.e., educational achievement during adolescence and educational attainment during the transition to adulthood and young adulthood) and young adults’ personal earnings [7]. Second, early socioeconomic adversity and genetic factors launch a life course *cascading process* of successively contingent educational achievements/failures over adolescence and the transition to adulthood [3,8]. Third, we posit that educational attainment at each life stage uniquely contributes to young adult educational and economic outcomes (hereafter referred to as a *cumulative process*) [7]. Previous research has not adequately investigated how additive, cascading, and cumulative processes relate to one another and condition one another, thus generating specific life course patterns and impacting youth outcomes.

Thus, the first objective of the present study is to investigate an integrated life course model [7], drawn from the life course theoretical perspective [9] to elucidate additive, cascading, and cumulative life course processes as well as potential interactions between genetics and socioeconomic environment (GxE) associated with young adult economic outcomes. In the present study, GxE interaction describes how early socioeconomic adversity may moderate (i.e., weaken or strengthen) the effects of genetic additive processes on educational outcomes at each life stage. The second objective is to examine the independent, varying associations among social and genetic predictors and their consequences for life-stage specific educational outcomes and young adult economic outcomes.

The present study uses data from 5,728 youth of European ancestry from The National Longitudinal Study of Adolescent to Adult Health (Add Health), which is a cohort study of youth who were in middle or high school in 1995 and were young adults by 2008. The hypothesized life course processes are described in the paragraphs that follow.

### Additive and cascading processes stemming from early socioeconomic adversity

Because family and community adverse socioeconomic circumstances often co-occur, creating cumulative adversity, children and adolescents frequently contend with constellations of developmental risk factors rather than isolated instances of adverse events [2]. Moreover, previous research has noted that exposure to multiple socioeconomic adversities in childhood and/or adolescence generally produces more severe consequences for youth development, particularly educational outcomes, than exposure to a single socioeconomic adversity [1–3]. Early socioeconomic adversity can curtail adolescents’ educational performance and undermine their psychosocial adjustment from adolescence into young adulthood.

Socioeconomically disadvantaged families are, broadly, families under stress [10], and stressful family environments are known to disrupt youth development and psychosocial functioning [11,12]. The accumulation of these risks results in biobehavioral system disruptions that are associated with childhood/adolescent cognitive, behavioral, and physiological impairments, including educational failures, poor mental health, and behavioral problems. These risks often continue into young adulthood [10,13]. Furthermore, socioeconomic adversity is connected to a general lack of educational and health resources, which may also contribute to youth academic problems [14,15]. Building on this research, the present study utilizes a composite index of multiple indicators capturing early cumulative socioeconomic adversity, including lower parental education, parents’ unstable marital history, family economic hardship, and adverse community characteristics. Educational outcomes across the early life course are conceptualized as academic performance or achievement (i.e., grade point average; GPA) in adolescence and educational attainment (i.e., number of years of formal education) in the transition to adulthood and young adulthood.

These additive associations between early socioeconomic adversity and educational outcomes may operate through social processes that differ across life stages [14–19]. For instance, educational performance in adolescence is primarily associated with family processes [19], whereas educational attainment in later stages (e.g., young adulthood) is more strongly associated with the availability of instrumental and financial support from the family of origin [3].

We posit that over and above additive processes, exposure to disadvantaged social conditions, such as family socioeconomic adversity, may initiate a cascade of educational failures [3,8]. That is, experiencing socioeconomic adversity in early life links to life-stage specific education failures in a successively contingent way, forming a “chain of insults” with increasingly negative impacts on educational outcomes across life stages that continues into young adulthood. Accordingly, youth from families with more socioeconomic adversity may experience education failures earlier in life and experience more rapid and successive failures in educational outcomes over the early life course compared than youth from families with less adversity. This is consistent with previous research has documented that adverse life experiences during adolescence can either build a solid foundation for later life or create “irreversibilities” that limit young adults’ opportunities and life chances [20].

### Additive and cascading processes stemming from genetic factor (education PGS)

In the present study, an education-related genetic factor was measured using a PGS for educational attainment. A PGS is a summary score for an individual based on the strength of the association between a set of genetic variants (i.e., single nucleotide polymorphisms, SNPs) and the outcome of interest (further discussed in the measurement section) [21]. PGSs with acceptable predictive validity have been developed for several outcomes including, body mass index (BMI), psychiatric disorders, smoking, and educational attainment [21–23]. However, it should be noted that many PGSs do not capture the full contribution of common genetic factors associated with the phenotype expected to be predicted. This may be attributed to the fact that the allelic (SNP) weights used to generate PGSs are estimated in independent finite samples, and therefore, contain some degree of measurement error.

#### Additive process stemming from education PGS

A number of studies have shown that education PGS is significantly associated with youth’s educational attainment regardless of their ancestry [5,24–27]. As shown in the left part of Fig 1, we expect that the education PGS is additively and uniquely associated with youth educational outcomes at each life stage (i.e., educational achievement in adolescence and educational attainment in the transition to adulthood and young adulthood). Several cognitive, behavioral, and emotional characteristics may be responsible for these genetic influences on educational outcomes. For example, characteristics such as higher cognitive aptitude, intelligence, mastery, self-control, and interpersonal skills as well as brain development are linked to genetics and contribute to positive educational outcomes [4,5,21,23,27–29]. Youth with these characteristics are likely to excel academically at each life stage. However, previous studies have not adequately examined life-stage specific genetic influences (G) on educational outcomes over the early life course.

Further, recent study findings suggest that PGS prediction accuracy, even in a single ancestry group with negligible causal allele frequencies, may vary depending on individual characteristics [30]. That is, PGS prediction accuracy depends on characteristics such as the socioeconomic status, age, or sex of the individuals in which the genome-wide association study (GWAS) was conducted [30]. Researchers suggest that this variation is attributed to the fact that different sets of genetic variants may contribute to phenotype prediction depending on the characteristics of study respondents [30]. Applying these findings to the present study, we expect that the genetic (i.e., PGS) influence on educational outcomes may vary across life stages as youth’s characteristics (e.g., age) and transitional events/status change. We argue that youth’s individual characteristics include their life-stage specific circumstances and needs. Thus, there is a possibility that the variation in the magnitude of the education PGS may also be attributed to the differences in education related to specific intra-individual processes at each life stage. In early life stages, education polygenic tendencies may be particularly important for the development of brain areas responsible for education. For example, in a previous study, an education PGS was associated with cortical total surface area and regions important for language and memory, which are necessary for educational achievement [28].

In the transition to adulthood and early adulthood, specific cognitive skills, such as advanced planning of curriculum decisions and persistence, are salient for academic degree completion (e.g., high school, college) [5,31]. Previous studies have shown a substantial genetic influence for both completing high school education and students’ choice of subjects [32]. Particularly, in early adulthood, positive characteristics, such as academic aspirations, may be more salient for promoting academic options [8]. Youth with greater academic aspirations may be more likely to seek out educational opportunities beyond high school.

In addition, previous studies [e.g., 30] suggest that the magnitude of the additive genetic influence on a phenotype is also determined existing indirect effects. Applying this notion to the present investigation, there may be a direct influence of the education PGS on educational attainment in young adulthood through educational attainment in the transition to adulthood. These indirect effects align with the hypothesized cascading process in the present study, enabling an investigation of unique, additive genetic influences on life-stage specific educational outcomes (additive process) after accounting for indirect effects. That is, the delineation of indirect effects in the hypothesized model in Fig 1 allows us to capture varying, additive genetic influence across life stages.

#### Cascading process stemming from education PGS

We posit that individuals with a higher education PGS will show greater educational achievement in adolescence over and above PGS additive processes, and they may have greater subsequent attainment through a cascading process. Thus, these individuals may experience academic acceleration over the early life course compared to individuals with a lower education PGS [33]. This cascading process is supported by the fact that there may exist multiple phenotypes, which are genetically correlated, with one phenotype influencing others through a mediational process (i.e., a mediated pleiotropy) [34]. For example, educational achievement in adolescence and educational attainment during the transition to adulthood can be considered as distinct phenotypes, which are genetically correlated forming a mediation process [34]. The hypothesized cascading process of educational outcomes across life stages reflecting this mediated pleiotropy is depicted in Fig 1.

### Gene-Environment Interaction (GxE)

In addition to the direct effects of genetic variants on educational outcomes, studies have found variants of genes often interact with environmental contexts to shape individuals’ psychological vulnerability and academic and cognitive competency [35]. In a meta-analysis that included several countries, Tucker and Bates [36] found support for a moderately-sized interaction between genetic variation and socioeconomic status. That is, the influence of genetic factors on educational attainment is filtered, altered, and shaped by socioeconomic context [21]. Therefore, we expect that the beneficial genetic influence on educational outcomes may be weakened by family socioeconomic adversity (GxE). That is, the education PGS is expected to predict educational outcomes, particularly for those with less cumulative adversity; in comparison, the education PGS may be less influential for those with more cumulative adversity. This expectation is consistent with the environmental bottleneck hypothesis, which suggests that adverse socioeconomic environments may limit the benefits of productive genes [4,37]. However, extending the notion of the variation in the genetic influences [30] to GxE influences, we expect that the weakening of life-stage specific genetic influences on educational outcomes due to family socioeconomic adversity may be distinct, or differentially vulnerable, across life stages.

### Independent associations among socioeconomic adversity, education PGS, and youth outcomes

Parents and offspring share some of their genetic dispositions [38] as genetic variation is inherited from parents. Also, the influence of family socioeconomic adversity on children’s educational attainment seems to be heritable as children from families with more socioeconomic attainment tend to have a greater genetic educational endowment [24,38]. However, as previously noted, education PGS may not capture the full contribution of common genetic factors associated with educational outcomes and may contain information about social environment context [39]. Despite these potential confounding or contamination of measures, in the present study, we expect that early socioeconomic adversity (i.e., comprehensive cumulative index) and youth genetic factors (i.e., education PGS), at least partly reflect contributions of genetics and social contexts to outcomes of youths which is their social mobility within their own life course [40].

### A cumulative process linking life-stage specific educational outcomes to economic outcomes

Education develops the competencies, skills, and behaviors necessary for socioeconomic attainment, including dependability, judgment, motivation, and effort [41]. Furthermore, educationally competent youth may more effectively utilize family economic resources (e.g., family income) and noneconomic resources (e.g., parental education and family relationships/support) leading to a successful transition to young adulthood [3]. Conversely, a lack of educational attainment and poor academic performance may lead to an unsuccessful transition to young adulthood, particularly in the form of more economic difficulties. More broadly, educational attainment at each life stage may have unique influences on the development of competencies, skills, and behaviors necessary for socioeconomic attainment in adulthood through various social and psycho-cognitive processes. Thus, as shown in Fig 1, we posit that life-stage specific educational attainment will uniquely influence economic outcomes in young adulthood. That is, at each life stage (i.e., adolescence, the transition to adulthood, young adulthood), educational attainment may enhance youth’s competencies, thereby increasing their likelihood of economic success in young adulthood. Previous research has rarely investigated the unique contributions of life-stage specific educational outcomes toward economic success in young adulthood.

### Hypotheses

The study hypotheses are as follows:

1. As an *additive* process, early socioeconomic adversity and education PGS are uniquely associated with life-stage specific educational outcomes over the first half of the life course (adolescence, transition to adulthood, and young adulthood) and young adults’ economic outcomes, as measured by their personal earnings.
2. A *cascading* process stemming from early socioeconomic adversity and genetic endowment links educational outcomes over the first half of the life course (adolescence, transition to adulthood, and young adulthood) leading to economic outcomes in young adulthood.
3. As a *cumulative* process, life-stage specific youth educational outcomes uniquely contribute to economic outcomes in young adulthood, as measured by their personal earnings.
4. Socioeconomic adversity moderates the association between education PGS and youth’s life-stage specific educational outcomes (GxE).

## Method

### Sample and procedure

Data for this study came from the National Longitudinal Study of Adolescent to Adult Health (Add Health; http://www.cpc.unc.edu/projects/addhealth), which is a nationally representative sample of middle and high school students at the first measurement occasion in 1995. Baseline (Wave 1) data were derived from a complex stratified cluster sampling of adolescents, yielding 20,745 respondents (mean age = 15.5 years; range 12 to 19 years at baseline and 25 to 32 years at Wave 4) from 134 middle and high schools. The sample was stratified by school region, urbanicity, type (public/private), racial composition, and size. The second, third, and fourth waves of data were collected in 1996, 2001, and 2008, respectively (N2 = 14,738; N3 = 15,100; N4 = 15,701).

As part of the Wave 4 data collection, saliva samples were obtained from consenting participants (96% of Wave 4 respondents). Genome-wide genotyping from saliva samples was conducted for approximately 80% of Wave 4 participants. Two Illumina platforms were utilized for genotyping (Illumina Omni1-Quad Bead Chip and Illumina Omni2.5-Quad Bead Chip). After quality control procedures, genotyped data were available for 9,974 individuals (7,917 from the Omni1 chip and 2,057 from the Omni2 chip) on 609,130 SNPs that are found on both genotyping platforms [42]. For more information on the genotyping and quality control procedures, see the Add Health GWAS QC report online at: http://www.cpc.unc.edu/projects/addhealth/documentation/guides/copy_of_AH_GWAS_QC.pdf.

Because the educational PGS was generated using a European ancestry sample, the present study was limited to a sub-sample of 5,728 youth of European ancestry. Further, researchers have noted that to account for population stratification analyses should control for ancestry-specific principal components. Following the Add Health recommendation, the present analysis included the first 10 principal components as control variables (https://www.cpc.unc.edu/projects/addhealth/documentation/guides/PGS_AH1_UserGuide.pdf).

Nearly 16% of data were missing for GPA and the early cumulative adversity measure. Missing values percentages for all the other study variables were less than 3%. Missing data were accounted for using Full Information Maximum Likelihood (FIML) procedure assuming missingness at random (MAR) [43].

### Measures

#### Early socioeconomic adversity

A composite index for early socioeconomic adversity was constructed by summing standardized measures to capture multiple dimensions of early family adversity. Higher scores of this composite measure reflect the greater severity of adversity [16].

*Parental education*. Mothers’ and fathers’ educational levels were summed to create an index of parental education. Responses ranged from 1 = *never went to school* to 10 = *beyond 4-year college degree*.

*Economic hardship*. At Wave 1 (1995), an index representing economic hardship was created by summing five dichotomous items (0 = *no* and 1 = *yes*) that assessed whether any member of the household received the following social service benefits in the past month: social security, supplemental security income, aid to families with dependent children, food stamps, or housing subsidies.

*Parents’ short marital duration*. To create an indicator of parents’ short marital duration, the number of years that parents were consistently married or in a marriage-like relationship (as reported by mothers in 1995) was subtracted from 20 (reported maximum duration). Higher values indicate a shorter marital duration and more time the child spent without consistently married parents.

*Community adversity*. Four indicators corresponding to census tract information from the 1990 U.S. Census were summed. The indicators included the proportion of (a) families living in poverty, (b) single-parent families, (c) adults employed in service occupations, and (d) unemployed men.

#### Educational outcomes

Educational outcomes were measured at three time points, capturing educational achievement in adolescence (1995) and educational attainment during the transition to adulthood (2001) and young adulthood (2008). For adolescent educational achievement, at Wave 1 (1995), respondents reported their recent letter grades in school mathematics, social studies, and science classes. These letter grades were based on a 4-point numerical scale (1 = D, 2 = C, 3 = B, and 4 = A). Sum scores were computed, with higher scores indicating higher academic performance. The number of years completed or highest grade/degree completed by the interview was used to indicate educational attainment for Wave 3 (2001, capturing the transition to adulthood) and Wave 4 (2008, capturing young adulthood). Response options varied from Wave 3 (ranged from 6 to 20) to Wave 4 (ranged from 1 to 13), but the response options were recoded to create identical categories (1 = less than high school, 2 = high school completed, 3 = some college, 4 = college degree, 5 = post graduate education).

#### Polygenic Score (PGS) assessing genetic educational endowment

GWASs have identified a large number of genetic variants (single-nucleotide polymorphisms, SNPs) that individually make very small but potentially meaningful contributions to phenotypes [44]. Researchers aggregate these SNPs across the entire genome with small effects to create a PGS that represents the aggregated genetic influence for various phenotypes of interest [45] and use the PGS in quantitative analyses [46]. For the specific phenotype of interest (e.g., educational attainment), regression coefficients for each SNP from an independent GWAS were used to weight the effects of SNPs for computing the education polygenic scale. Accordingly, the PGS represents the aggregated genetic influence of a large number of SNPs that are associated with educational attainment. It was calculated from the Add Health GWAS study [47], following the procedure outlined in Dudbridge’ study [48]. Information on the SNPs used to generate the education polygenic scale can be found in the previous study [23]. The raw PGSs were standardized within ancestry groups to account for between-group population stratification. More details can be found at https://www.cpc.unc.edu/projects/addhealth/documentation/guides/PGS_AH1_UserGuide.pdf.

#### Young adult economic outcomes

At Wave 4 (2008), young adults’ personal earnings were used as an indicator of their economic outcomes. Young adults reported their annual personal *earnings* before taxes (including wages/salaries, tips, bonuses, overtime pay, and self-employment income) for 2006, 2007, and 2008, and an average was computed.

### Analytic plan

We used a structural equation model (SEM) to test the hypothesized model. SEM allows for several multiple regression analyses to be tested together within the same analytical model as a path analysis representing life course processes. Accordingly, an SEM may contain multiple exogenous (i.e., predictor) variables and multiple endogenous (i.e., dependent) variables, including mediators and outcomes. By adding variables and paths to the basic model in a meaningful manner based on study hypotheses, a series of incremental models can be fit that culminate in the development, and analysis, of a comprehensive model. In the present study, a series of such incremental SEMs were estimated. First, Model 1 was estimated to target the additive and cascading processes beginning from early socioeconomic adversity and cumulative educational process. Next, Model 2 incorporated the education PGS along with the 10 ancestry-specific principal components into Model 1 to consider how education PGS and socioeconomic adversity independently contribute to educational and economic outcomes. Model 2 also incorporated a product term to test for GxE interactions between education PGS and socioeconomic adversity. Gender and age were included as control variables in all models. Standardized coefficients are reported as effect sizes (*r*-matrix) [49].

Because the allelic weights of the education PGS were generated using a sample of individuals of European ancestry, education PGS may be less predictive for other ancestry groups [50], only European ancestry group was used in the study.

A range of fit indices was used to evaluate model fit, including the chi-square statistic, Cumulative Fit Index (CFI), and Root Mean Square Error of Approximation (RMSEA). The model is thought to fit the data well when the chi-square divided by the degrees of freedom is below 3.0 [51]. CFI values near or greater than .95 and RMSEA values close to or less than .06 indicate that the model fits the data well [52]. Mplus software, version 8.00 was used for all analyses [53]. Potential clustering of data was accounted for using TYPE = COMPLEX command in Mplus.

## Results

Descriptive statistics and correlations among study variables are shown in Table 1. Correlations among study variables were statistically significant and in the expected direction except for the correlation between age and EDU1(GPA). This negative association warrants further investigation. PGS education, early socioeconomic, age, and gender adversity were significantly correlated with young adults’ personal earnings (2008).

**Table 1:** Correlations, Means, and Standard deviations among study variables.

	1	2	3	4	5	6	7	8
1. ESA *(1995)*	−							
2. EDUPGS	-.15***	−						
3. EDU1 (GPA)*(1995)*	-.21***	.16***	−					
4. EDU2 *(2001)*	-.36***	.20***	.30***	−				
5. EDU3 *(2008)*	-.36***	.24***	.37***	.66***	−			
6. P. earnings (*2008)* (in thousands)	-.21***	.10***	.15***	.31***	.27***	−		
7. Female ^*a*^	.03*	-.02	.07***	.08***	.12***	-.20***	−	
8. Age *(1995)*	.01	-.03	-.23**	.31**	.40**	.17**		−
*Mean or %*	-.10	0.00	8.67	13.25	5.62	39.28	52.71%	15.1
*SD*	3.50	1.00	2.81	1.97	2.15	25.83	.50	1.9
*Maximum*	18.10	3.55	14.00	22.00	30.00	150.00	1	20.0
*Minimum*	-7.28	-4.13	1.00	6.00	1.00	2.50	0	10.2

Note. a = Reference is Male. ESA = Early Socioeconomic Adversity. EDUPGS = Education Polygenic Score. EDU1 = Educational achievement in adolescence. EDU2 and EDU3 = Educational attainment in the transition to adulthood and young adulthood, respectively. P = Personal.

### Socioeconomic adversity, educational outcomes, and economic outcomes (Model 1)

**Fig 2:**
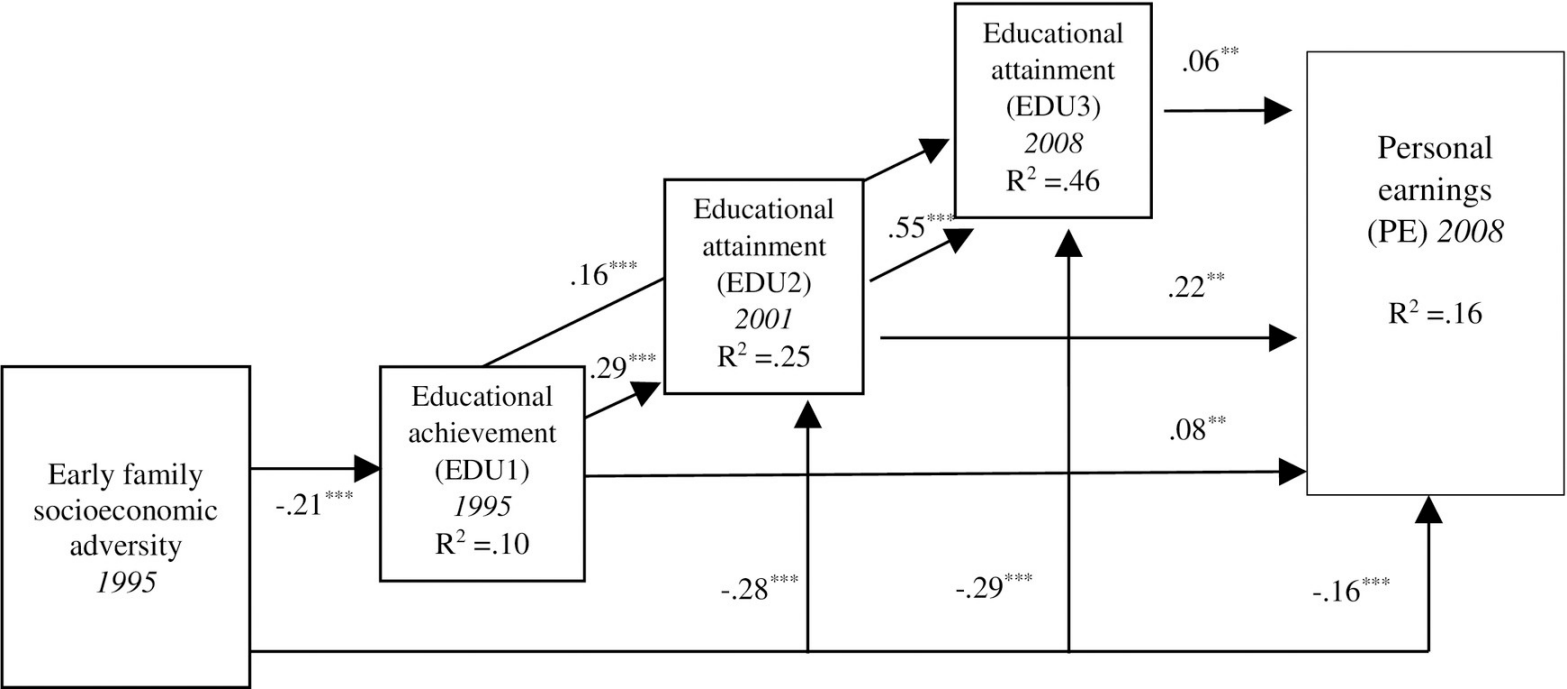
Model 1: Additive, cascading, and cumulative processes stemming from family socioeconomic adversity. Notes. Age was significantly associated with Ed1, Ed2, Ed3, and PE (-.23, .30, .16, and .06, respectively, *p* < .05). Gender (female) was significantly associated with EDU1, EDU2, EDU3, and PE (.06, .10, .06, and-.25, respectively, *p* < .05). Standardized coefficients are shown. **p < .01. ***p < .001. *χ*^2^(2) = 3.05, CFI = .99, RMSEA = .03, *p* < .001. CFI = 1.00. RMSEA = .00.

The first model (i.e., Model 1 shown in Fig 2) incorporated respondents’ socioeconomic adversity, their educational outcomes at three life stages (adolescence, the transition to adulthood, and young adulthood; Waves 1, 3, and 4, respectively), and their personal earnings as young adults (Wave 4, 2008). Providing evidence for an additive process, socioeconomic adversity was negatively associated with educational outcomes in adolescence (Wave 1: *β* = -.21, *p* < .001), the transition to adulthood (Wave 3: *β* = -.28, *p* < .001), and young adulthood (Wave 4: *β* = -.29, *p* < .001). In turn, the educational outcome at each life stage influenced young adult earnings (*β* = .08, .22, and .06 for educational outcomes at Waves 1, 3, and 4, respectively). Socioeconomic adversity was also directly associated with young adults’ earnings (*β* = -.16, *p* < .001) after adjusting for the effects of educational outcome at each life stage. In addition, providing evidence for a cascading process, longitudinal associations existed between educational outcomes over the early life course. For example, educational achievement in adolescence (Wave 1) was linked to educational attainment during the transition to adulthood (Wave 3) (*β* = .29, *p* < .001). Similarly, educational attainment during the transition to adulthood was associated with attainment in young adulthood (Wave 4) (*β* = .55, *p* < .001). Educational achievement at Wave 1 also directly influenced Wave 4 educational attainment (*β* = .16, *p* < .001). Age is associated with educational outcomes at each life stage and young adult earnings (*β =* -.23, .30, .16, and .06, *p* < .05, respectively). Gender also influenced educational outcomes at each life stage and young adult earnings (*β* = .06, .10, .06, and -.25, *p* < .05, respectively).

### Adding education PGS (Model 2)

**Fig 3:**
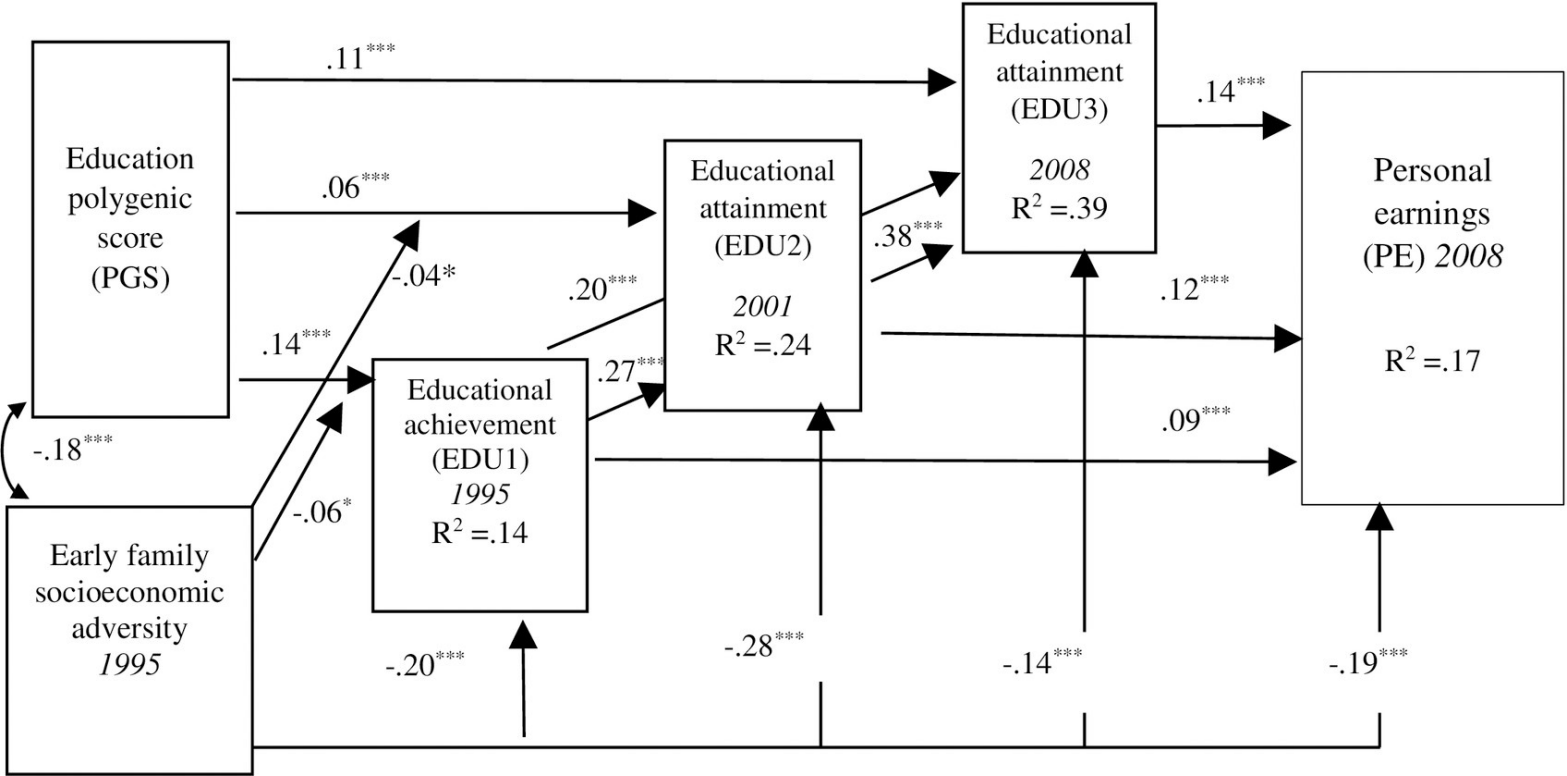
Model 2: Additive, cascading, and cumulative life course processes from family socioeconomic adversity and PGS. Note. Age was significantly associated with ED1, ED2, ED3, and PE (-.21, .27, .20, and .07, respectively, p < .05). Gender (female) was significantly associated with EDU1, EDU2, EDU3, and PE (.07, .11, .08, and -.25, respectively, p < .05,). Standardized coefficients are shown. Non-significant paths are not shown. *p < .05. ***p < .001. p < .001. CFI = .96. RMSEA = .02.

Model 2 (shown in Fig 3) incorporated the education PGS as a predictor of educational outcomes at the three life stages and young adult personal earnings. The education PGS was independently and additively associated with educational outcomes at all three life stages with varying magnitudes (*β* = .14, *β* = .06, and *β* = .11, *p* < .001, for Waves 1, 3, and 4, respectively). There was a negative association between socioeconomic adversity and the education polygenic scale (*r* = -.18, *p* < .001). That is, respondents with more education-related genetic variants generally experienced less socioeconomic adversity at Wave 1. In addition, all of the associations involving socioeconomic adversity from Model 1 remained statistically significant, indicating unique associations between socioeconomic adversity and educational outcomes at all three life stages after accounting for the education PGS. For example, those with more socioeconomic adversity reported less educational outcomes at Waves 1, 3, and 4 (*β* = -.20, .28, and -.14, *p* < .001, respectively). Also, socioeconomic adversity was negatively related to young adults’ earnings (*β* = -.19, *p* < .001). Age is associated with educational outcomes at each life stage and young adult earnings (*β* = -.21, .27, .20, and .07, *p* < .05, respectively). Gender also influenced educational outcomes at each life stage and young adult earnings (*β* = .07, .11, .08, and -.25, *p* < .05, respectively).

**Fig 4:**
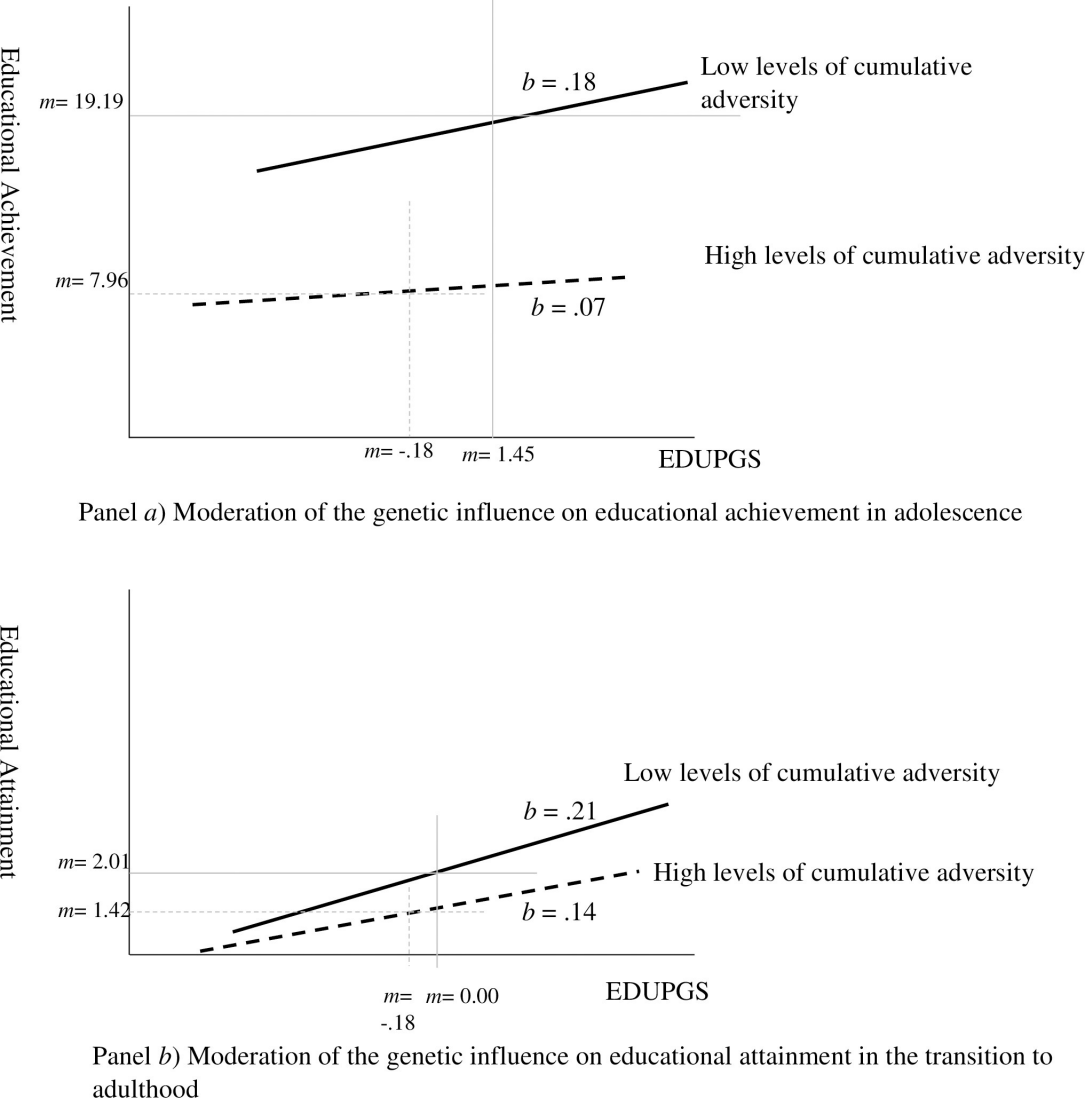
Moderation of the genetic influence on educational outcomes by early socioeconomic adversity (G×E). Note. *b* = Unstandardized slope coefficients. M = Mean. EDUPGS = Education Polygenic Scale.

Varying GxE effects were found between socioeconomic adversity and education PGS for educational outcomes at two life stages, namely adolescence (*β* = -.06, *p* < .05) and the transition to adulthood (*β* = -.04, *p* < .05). The interactions are depicted in panels A and B in Fig 4. These interactions suggest a bi-linear association between socioeconomic adversity in adolescence and youth outcomes, depending on their level of education PGS.

## Discussion

Although recent research suggests that individual socioeconomic position and genetics can be fundamental contributors to life outcomes [5,13], less is known about how early socioeconomic adversity and genetic factors are independently and jointly associated with youth socioeconomic outcomes over the life course. Thus, the present study examined life course processes linking early socioeconomic adversity and individual genetic factors (assessed by a PGS) to educational outcomes at three life stages (educational achievement in adolescence and educational attainment in the transition to adulthood and young adulthood) and, in turn, to young adults’ economic outcomes, as measured by personal earnings.

First, the results supported our expectation that early socioeconomic adversity is additively and independently associated with educational outcomes at each life stage. The strength of this association was relatively consistent across all three life stages, despite the variation in educational outcome assessed from adolescence to later life stages. These additive influences suggest that structural socioeconomic constraints and adverse family processes associated with early socioeconomic adversity influence educational outcomes throughout the early life course. Given that most previous developmental research focusing on the influence of socioeconomic adversity has not investigated such additive influences across developmental stages, these findings enhance our understanding of the persistent association between early socioeconomic adversity and youth socioeconomic outcomes over the life course. However, future studies should elucidate socioeconomic and family processes responsible for these influences that may differ across life-stages.

In addition, early socioeconomic adversity was linked to later educational outcomes in the transition to adulthood and young adulthood in a chain-like manner, with impacts for subsequent economic outcomes in young adulthood. As a cascading process, these results suggest that the association between early impairments in educational outcomes and later economic outcomes continues over the life course [8] and the association is not spurious due to the common influence of early socioeconomic adversity. Moreover, this cascading process may develop cumulative disadvantage characteristics within individuals because early impairment often limits an individual’s ability to acquire necessary resources and services, which increases the likelihood of further impairments in their socioeconomic achievements [30]. A deeper understanding of cascading effects could aid in breaking this "chain of insults" [8].

This cascading process also further emphasizes the salience of adverse socioeconomic experiences in adolescence. This early influence is consistent with the ‘*critical period’* notion, which posits that exposure to early socioeconomic adversity during adolescence has crucial effects and causes irreversible damages, independent of later life experiences. These damages may include impaired psychological and biological development, which, in turn, can have detrimental impacts on educational outcomes [14]. Thus, these findings highlight early educational success, as reflected by early educational performance (e.g., adolescent GPA), as a prime focus for policies and interventions addressing youth development.

Educational outcomes at all three life stages additively contributed to economic success in young adulthood, signaling the formation of comprehensive, cumulative developmental success (or risk) exposure for young adults. Particularly, the results showed that adolescents’ educational achievement was directly associated with their educational and economic attainments as young adults after accounting for lagged proximal educational attainments. This is consistent with the “critical period” notion, which emphasizes adverse experiences and associated early “damages” can have persistent independent influences [14]. In contrast, early educational outcomes may develop essential competencies for socioeconomic success in young adulthood [41]. Together, these findings further highlight the importance of addressing early educational outcomes in policies and programs targeting positive youth development.

In addition, the findings suggest that educational outcomes in later life are directly related to personal earnings, regardless of early educational success or failure, emphasizing the additive contribution of later educational attainment. This may be attributed to youth experiencing positive (or negative) “turning points” in later stages (e.g., increased family/spousal/partner support or receiving financial support), which may contribute to later educational success and, in turn, personal earnings. Thus, the transition to adulthood remains an important life stage for intervention programs that prepare individuals to succeed in young adulthood and throughout adulthood.

The second model showed that both family socioeconomic adversity and youth’s education PGS additively and independently contributed to educational outcomes at all three life stages after accounting for gene-environment correlations. There was variation in the strength of the additive genetic associations (G) between life stages, suggesting that this variation may be attributed to life-stage specific individual characteristics, such as age and individuals’ transitional circumstances/needs [30]. Thus, genetics may influence educational outcomes through life-stage specific biological (e.g., biological encoding, brain development and functions such as neuron communications) [29] and cognitive processes [25] that are relevant to educational outcomes at each life stage.

In addition, as with socioeconomic adversity, youth’s genetics also appear to trigger cascading educational process leading to their earnings as young adults [8]. This cascading process links life-stage specific educational outcomes (educational achievement and attainment, considered as two phenotypes), which are genetically correlated, and forms a longitudinal mediational process (i.e., mediated pleiotropy) [34]. Thus, the link between successive educational outcomes does not appear to be spurious (i.e., biological pleiotropy) [34] and due to the common genetic influence. Instead, genetic tendencies related to early educational development operate over the life course. As previously noted, these findings highlight (a) the need to strengthen the cascading process of educational success while breaking the cascading process of educational failure over the early life course. Findings also suggest that early (i.e., adolescent) educational achievement may be more malleable and, thus, should be a prime focus of developmental interventions because it appears to be a necessary condition for the initiation of educational attainment process.

In sum, the findings based on the integrated life course model demonstrated how additive, cascading, and cumulative processes relate to and condition one another, generating specific life course patterns and outcomes. A notable cascading process in the results represents indirect genetic influences on educational outcomes, which may influence the magnitude of additive genetic effects on educational outcomes [30]. However, unique, additive genetic influences were noted at each life stage, indicating that life-stage specific genetic tendencies are also important for youth’s educational development over the life course. These life-stage specific tendencies may contribute to “turning points” in the lives of youth. Future studies should further investigate potential genetic influences on turning points in youth life trajectories.

Furthermore, youth’s education PGS interacted with their early socioeconomic adversity (GxE) to influence their educational achievement in adolescence and attainment in the transition to adulthood. These interactions can be interpreted as the weakening of the positive polygenic influence on educational outcomes by an adverse socioeconomic context. Multiplicative effects between early adversity and educational outcomes, particularly for educational failures, is also consistent with the cumulative disadvantage notion [33] and illustrates how increasing inequality in educational outcomes results over time. However, the interaction was not significant in young adulthood, which is evidence to show that the GxE effect on educational outcomes varies across life stages. These variations may be due to youth’s changing socioeconomic context, which occurs parallel to their life transitions and circumstances, such as marriage and work [54]. This variation in GxE moderation depending on individual characteristics of respondents (i.e., life stage) is parallel to the notion of variation in genetic influence (G) [30]. Studies should further investigate if, and how, individual genetic influence varies across different cultures and across other life stages.

Several factors potentially limit the scope and generalizability of the results. First, as noted earlier, education PGS may not capture the full genetic contribution to youth educational outcomes. Second, the contribution of education PGS to youth outcomes may not be unique (i.e., independent) from early socioeconomic adversity because the PGS may already contain environmentally mediated effects of parental genotypes (i.e., “genetic nurture”) [37]. Third, the present study used self-report measures of educational outcomes and personal earnings. Replication using various objective and independent reports (e.g., school records, tax returns) would alleviate concerns regarding potential self-report biases. Fourth, standardized tests may be better measures of educational achievement than GPA as GPA calculations may differ across schools. Fifth, other potential confounding variables (e.g., physical health, neurological factors) were not included in the present study. Finally, recent studies have suggested that the influence of early socioeconomic adversity on educational outcomes may still include some variation that is linked to parents’ genetics, because the influence of parents’ genetics on youth outcomes may partly be mediated by parents’ characteristics and the family socioeconomic environment [5,55].

In sum, the findings highlight the need to incorporate early socioeconomic and molecular genetic information into longitudinal life course developmental research. This calls for an integrated developmental life course model that elucidate how additive, cascading, and cumulative processes relate to and condition one another, thus generating specific life course patterns and socioeconomic outcomes over the early life course. The present study’s findings also reinforced suggestions of recent studies, highlighting the need to incorporate molecular genetic information into life course developmental research and revisit existing developmental and social science theories [4,5].
